# Monitoring changes in the genetic structure of Brown Tsaiya duck selected for feeding efficiency by microsatellite markers

**DOI:** 10.5713/ab.22.0213

**Published:** 2022-11-13

**Authors:** Yi-Ying Chang, Hsiu-Chou Liu, Chih-Feng Chen

**Affiliations:** 1 ILan Branch, Livestock Research Institute, Council of Agriculture, Executive Yuan 26846, Taiwan; 2 Department of Animal Science, National Chung Hsing University, Taichung 40227, Taiwan; 3 The iEGG and Animal Biotechnology Center, National Chung Hsing University, Taichung 40227, Taiwan

**Keywords:** Duck, Genetic Monitoring, Microsatellite Marker

## Abstract

**Objective:**

Few studies have genetically monitored chickens over time, and no research has been conducted on ducks. To ensure the sustainable management of key duck breeds, we used microsatellite markers to monitor Brown Tsaiya ducks over time genetically.

**Methods:**

The second, fourth, sixth to eighth generations of the Brown Tsaiya duck selected for feeding efficiency and control lines were included in this study to investigate the genetic variations, effective population size, population structure and the differentiation between populations over time with 11 microsatellite markers derived from Brown Tsaiya duck.

**Results:**

The results showed there were a slight decrease in the genetic variations and an increase in within-population inbreeding coefficient (*F*_IS_) in both lines, but no consistent increase in *F*_IS_ was observed in each line. The effective population size in the second and eighth generations was 27.2 for the selected line and 23.9 for the control line. The change in allele richness showed a downward trend over time, and the selected line was slightly lower than the control line in each generation. The number of private alleles (N_p_) in the selected line were higher than in the control line. Moderate differentiation was observed between the second and eighth generations in the selected line (*F*_ST_ = 0.0510) and the control line (*F*_ST_ = 0.0606). Overall, differentiation tended to increase with each generation, but genetic variation and structure did not change considerably after six generations in the two lines.

**Conclusion:**

This study provides a reference for poultry conservation and helps to implement cross-generation genetic monitoring and breeding plans in other duck breeds or lines to promote sustainable management.

## INTRODUCTION

The Brown Tsaiya (*Anas Platyrhynchos*), the only layer type duck breed in Taiwan, has a small body and a high egg-laying rate; their eggs are heavy and have strong shells. They are the primary source of processed eggs (preserved and salted eggs) in Taiwan. The feed costs account for over 80% of the total duck egg production cost. Because of continuous increases in the price of feed ingredients, to help farmers lower costs, the Livestock Research Institute in Taiwan began selecting low residual feed consumption (RFC) in 2009 [1] to improve the feed efficiency in laying ducks. The Individuals with lower RFC, originating from a high egg production line Brown Tsaiya LRI 1, were used as foundation stock (G0) of the selected line, while the other individuals as the control line. Data on RFC-related traits, including feed consumption, egg mass, and changes in body weight, were collected from more than 200 female ducks of multiple generations. Based on these data and the pedigree of individual ducks, estimated breeding values were calculated to select breeder ducks. After the selection for six generations, the line was named “Better Feed Efficiency Brown Tsaiya” in 2018. The laying performance of the line is identical to that of the control line, but its average feed consumption at 34 to 37 weeks of age is 10% lower than that of the control line. The selected line is still under selection and promoted to the industry through duck breeder farm.

The RFC selected and control lines were both closed populations of approximately 200 ducks in each generation, to avoid severe inbreeding, the candidate breeder ducks were divided into 12 sire families and preventing full- and half-sib mating. According to the guidelines of the Food and Agriculture Organization of the United Nations (FAO), in the conservation of animal genetic resources, in addition to recording basic information and appearance traits, to determine the effects of conservation and selection strategies on genetic heterozygosity, molecular markers should be also used to investigate the genetic structure [2]. Following the guidelines of FAO, we noted egg production, eggshell quality, and the growth traits for every generation in the two lines to monitor their performance. Besides, we use microsatellite markers to investigate the duck’s genetic structure.

Microsatellite markers have been widely used in studies on indigenous and commercial duck population conservation in India, the Philippines, Vietnam, China, and other countries [3–7] because its characteristics of multiple alleles per locus, widely distributed in eukaryotic genomes, not strongly affected by selection, rich in polymorphic information, and low cost [8–10]. However, although a few studies have investigated the genetic polymorphisms of duck breeds for conservation, most studies investigated multiple populations at a single time point; no cross-generational genetic monitoring study on ducks has been conducted. On the other hand, some cross-generation monitoring studies on chickens, and they pointed out that the smaller the population is, the more often their allele frequencies change, and monitoring changes in genetic polymorphisms over time is crucial for poultry conservation and breeding programs [11,12].

In our previous study, we used 11 Tsaiya microsatellite markers to investigate the genetic structure of various Brown Tsaiya duck lines, including the second and fourth generations of the RFC selected and control lines. Compared with the germplasm-preserved Brown Tsaiya duck (without selection), there was almost no change in genetic variations between the population separated by two generations, while RFC control line showed a decrease in the number of allele and heterozygosity [13]. Lai et al [14] also showed that compared with germplasm-preserved Brown Tsaiya duck, the Brown Tsaiya duck lines selected for specific traits had higher values in within-population inbreeding coefficient (*F*_IS_) and more markers deviating from Hardy–Weinberg equilibrium. Since the RFC selected line is still under selection, to ensure sustainable management, the aim of this study is to integrate our previous study and conduct genotyping in subsequent generations of the RFC selected and control line to evaluate the effects of continuous selection and breeding strategies on the genetic variation and differentiation over time.

## MATERIALS AND METHODS

### Animal care

The animals, management methods, and experimental protocols used in this study were approved by the Institutional Animal Care and Use Committee of the Ilan Branch of the Livestock Research Institute, Council of Agriculture, Executive Yuan, Taiwan (case numbers LRI IACUC 104-012, LRIIL IACUC107004, LRIIL IACUC108003, and LRIIL IACUC 109010).

### Sample collection and DNA extraction

The experimental animals were from 10 populations of the second, fourth, sixth, seventh, and eighth generations of the selected and control lines. The two lines’ genotypes of the second and fourth generations were obtained from the previous study (i.e., the RFC selected line and control line of the 18th and 20th generations of Brown Tsaiya LRI 1) [13], and the other generations were genotyped in this study. To increase the representativeness of the samples, we selected two males and two females from each sire family of the two lines. Table 1 presents the number of samples in each population.

### Microsatellite genotyping

For each duck in this study, 2 to 3 mL of blood were drawn from the superficial plantar metatarsal vein or wing vein. The genomic DNA of the ducks was extracted from the blood samples by removing plasma with a Gentra Puregene Blood Kit (Qiagen, Venlo, The Netherlands) under the instructions; we followed Bush et al [15] to adjust the amount of blood samples.

The microsatellite markers were screened from Brown Tsaiya ducks [16]. After testing several Tsaiya duck populations, we selected those with superior polymorphisms (Table 2), namely APT001, APT004, APT008, APT010, APT012, APT017, APT020, APT025, APT026, APT032, and APT033, for a total of 11 markers. For the microsatellite analysis, polymerase chain reaction (PCR) was performed using a Veriti 96 Well Fast Thermal Cycler (Applied Biosystems by Thermo Fisher Scientific, Waltham, MA, USA). A final volume of 10 μL solution containing 0.375 U of Taq DNA polymerase (TaKaRa, Kyoto, Japan), 1×PCR buffer (1.5 mM MgCl_2_), 0.2 μM deoxyribose nucleotide triphosphate (dNTP), 0.2 μM forward and reverse primers with fluorescent-labeled in the forward primer, and 50 ng of genomic DNA. The PCR cycling program was as follows: 94°C for 10 min, 30 cycles at 94°C for 20 s, 60°C for 30 s, 72°C for 30 s, and final elongation at 72°C for 10 min. The PCR products were analyzed through gel electrophoresis in 1% agarose gels, visualized using ethidium bromide staining, and pooled based on fragment size and fluorescent color for subsequent capillary electrophoresis analysis. The amplified microsatellite PCR products were denatured using Hi-Di formamide and analyzed using a DNA analyzer (ABI PRISM 3730 DNA analyzer; Applied Biosystems by Thermo Fisher Scientific, USA). Fragment size was estimated using GeneScan 500 LIZ Size Standard (Applied Biosystems by Thermo Fisher Scientific, USA). The resulting fragment analysis data and size of the alleles were analyzed using Peak Scanner Software version 1.0 (Applied Biosystems by Thermo Fisher Scientific, USA).

### Statistical analysis

The genetic variation data, which consist of the number of alleles (N_a_), observed (H_O_), and expected (H_E_) heterozygosity [17], and the polymorphic information content (PIC) [18], were calculated using Cervus (version 3.0) [19]. POPGENE 1.32 [20] was used to calculate the number of effective alleles (N_e_), and estimate Wright’s *F*-statistics for each locus, namely within-population inbreeding coefficient (*F*_IS_), among-population genetic differentiation (*F*_ST_), and population inbreeding coefficient (*F*_IT_), and to perform the Hardy–Weinberg equilibrium test [21] and Ewens-Watterson neutrality test to evaluate the selective neutrality of the 11 microsatellite markers [22]. We also used FreeNA [23] to detect the null allele of the 11 microsatellite markers. Effective population size (N_e_) was estimated using the Jordan–Ryman temporal method and plan I in NeEstimator (V2) [24] with consideration for the mating system and sampling. The temporal change in allele frequency was used to calculate the mean Ne at different sampling time points [25]. FSTAT (version 2.9.3) [26] was used to estimate the allele richness (AR) and pairwise *F*_ST_ between populations as described in Weir and Cockerham [27] to evaluate the degree of differentiation between populations. The analysis of molecular variance (AMOVA) and calculating the number of private alleles per locus (N_p_) were performed using GenAlEx (v6.503) [28] to divide the total genetic variance into population- and individual-based components. These parameters were used to investigate the markers’ polymorphisms and the change of genetic variation in the selected and control lines through the generations.

In the clustering analysis, the adegenet R package performed principal coordinate analysis (PCA) and plotting [29]. The clustering of individuals from multilocus genotypes was performed using the STRUCTURE (version 2.3) [30] admixture model. The number of clusters (K) was between 2 and 10. For each K, 100 independent runs were performed with 20,000 burn-in periods followed by 50,000 iterations. The optimal K was determined using STRUCTURE HARVESTER (v0.6.91) [31] following the Evanno method [32], and the results from STRUCTURE were integrated into CLUMPAK (version 1.1) [33] to visualize the long-term effects of selection on population differentiation.

## RESULTS

### Microsatellite marker polymorphism and *F*-statistics

Table 3 presents the polymorphisms and *F*-statistics of the 11 Tsaiya duck microsatellite markers in the selected and control lines. Fifty-four alleles were observed, and all 11 loci were polymorphic. The number of alleles (N_a_) ranged from 2 to 8, with an average of 4.9 alleles per locus. The average number of effective alleles (N_e_) per locus was 2.900 ranged from 1.911 (APT033) to 4.106 (APT004). The observed heterozygosity (H_O_) among 11 microsatellite loci had a range from 0.227 (APT033) to 0.758 (APT026), with the average of 0.531. The expected heterozygosity (H_E_) ranged from 0.477 (APT033) to 0.757 (APT004), with an average of 0.627. The PIC ranged from 0.363 (APT033) to 0.720 (APT026), with an average of 0.563. Among the 11 microsatellite markers, seven markers were highly polymorphic (PIC>0.5), and four markers were moderately polymorphic (0.5>PIC>0.25). Nevertheless, the Ewens-Watterson test indicates APT008 and APT026 are not selectively neutral (Supplementary Table S2). In addition, APT001, APT004, APT008, APT010, and APT017 deviated from the Hardy–Weinberg equilibrium (p<0.01). As for the *F*-statistics, the *F*_IS_ of the 11 microsatellite markers ranged from −0.097 (APT026) to 0.502 (APT033), with an average of 0.102. The *F*_IT_ ranged from −0.064 (APT026) to 0.538 (APT033), with an average of 0.154. The *F*_ST_ ranged from 0.021 (APT032) to 0.133 (APT010), with an average of 0.059. The results indicated that 5.9% of the overall genetic variation was due to differences between populations, whereas differences among individuals within each population caused 94.1% of total genetic variation.

### Inter- and intra-line comparison of genetic variation

Table 4 presents the average genetic variation of the 11 Tsaiya microsatellite markers in the various generations of the selected line (S) and the control line (C).

The inter-line comparison showed that N_a_ was higher in most of the generations of the selected line than in the control line; only S2 and S8 had slightly lower N_a_ values than did C2 and C8. N_e_, H_O_, H_E_, and PIC in the control line were higher than those in the selected line, except for the eighth generation. The mean AR values estimated based on the minimum sample size 17 ranged from 3.189 (S8) to 3.727 in selected line, while 3.545 (C8) to 3.777 (C6) in control line. The selected line is slightly lower than the control line in each generation, especially in the eighth generation. The number of private alleles (N_p_) ranged from 0.000 (S2) to 0.364 (S7) in selected line, and 0.000 (C4, C6, C7, and C8) to 0.091 (C2). Compared to the selected line, there was almost no private allele in the control line. However, except for Np no significant difference in genetic variation was observed between the lines (p>0.05).

Some markers deviated from the Hardy–Weinberg equilibrium in the sixth to eighth generations, namely APT001 and APT033 in the sixth generation of both lines, APT001 in S7, APT008 and APT033 in S8, and APT001 and APT008 in C8. *F*_IS_ was positive in all generations of both lines, but no difference between the lines was observed.

The intra–line comparison showed that the selected line, N_a_, N_e_, H_O_, H_E_, and PIC slightly increased then decreased with each generation, while those continued decreasing slight in the control line. The change in AR showed a downward trend over time. *F*_IS_ ranged from 0.082 (S2) to 0.137 (S7) in the selected line and from 0.048 (C2) to 0.165 (C4) in the control line, and no consistent increase was observed in each line. In addition, no significant differences in genetic variation were observed between generations in each line (p>0.05).

The effective population size (Ne) in the second and eighth generations, estimated through the Jorde–Ryman temporal method, was 27.2 for the selected line (95% jackknife confidence interval: 17.3 to 63.4) and 23.9 for the control line (95% jackknife confidence interval: 16.6 to 42.3); Ne was lower in the control line than in the selected line. We also estimated Ne in two generations of each line; the closer the two generations were, the higher the Ne was. For example, the upper limits of the confidence intervals in the seventh and eighth generations of the two lines were infinity (Supplementary Table S3).

The result of FreeNA [23] (Supplementary Table S4) showed that the null allele frequencies of the APT001 and APT033 in 3 and 1 populations were greater than 0.2, respectively, and the null allele frequencies of the two markers in 3 and 6 populations were between 0.15 and 0.2. The null allele frequencies of APT008 in the fourth and eighth generations of both lines were higher than 0.2.

### Population differentiation and genetic structure analysis

Table 5 presents a pairwise comparison of *F*_ST_ between generations of the selected line and its control line. The intra-line comparison revealed low *F*_ST_ values in the second and sixth generations: 0.0307 and 0.0217, respectively. Moderate *F*_ST_ values were observed in the fourth, seventh, and eighth generations: 0.0540, 0.1123, and 0.1271, respectively. *F*_ST_ gradually increased from the second to fourth generations, decreased in the sixth generation, and increased again to 0.1123 in the seventh generation and to 0.1271 in the eighth generation. Overall, differentiation tended to increase with each generation.

The intra-line comparison in the selected line showed that S8 was significantly differentiated from S2, S4, and S6, and the *F*_ST_ values range from 0.0074 to 0.0510. However, only moderate differentiation was observed between S2 and S8 (*F*_ST_ = 0.051). Significant differentiation was observed between C2 and C7 to C8 and between C8 and C4 to C6, with *F*_ST_ values ranging from 0.0240 to 0.0606. Only moderate differentiation was observed between C2 and C8 (*F*_ST_ = 0.0606). The results indicate that *F*_ST_ increased as the generation gap widened for both the selected and control lines.

The AMOVA results in Table 6 indicate that largest variation in the second and eighth generations were within individuals, accounting for 92% and 76%, respectively, followed by variation among individuals, accounting for 5% and 12%. The variation among populations accounted for 3% and 12%, respectively. The results also indicate that the proportion of genetic variation among individuals and among populations increased between the second and eighth generations and that the eighth generation had a higher proportion of genetic variation than the second.

Population PCA was performed using the adegenet R package (Supplementary Figure S1). The percentage of variation explained by the first, second, and third axes was 8.5%, 5.1%, and 4.4%, respectively. Because we focused on the cross-generation analysis of two lines, the proportion explained by PCA was low. Because of the low defined proportion, we plotted the first and second principal coordinates. Although the selected line and its control line were distributed into two groups in the PCA, numerous overlapping sections were observed. No significant differences in distribution range were observed among generations, indicating that the population structure was similar and that polymorphisms did not decrease.

**Figure 1:**
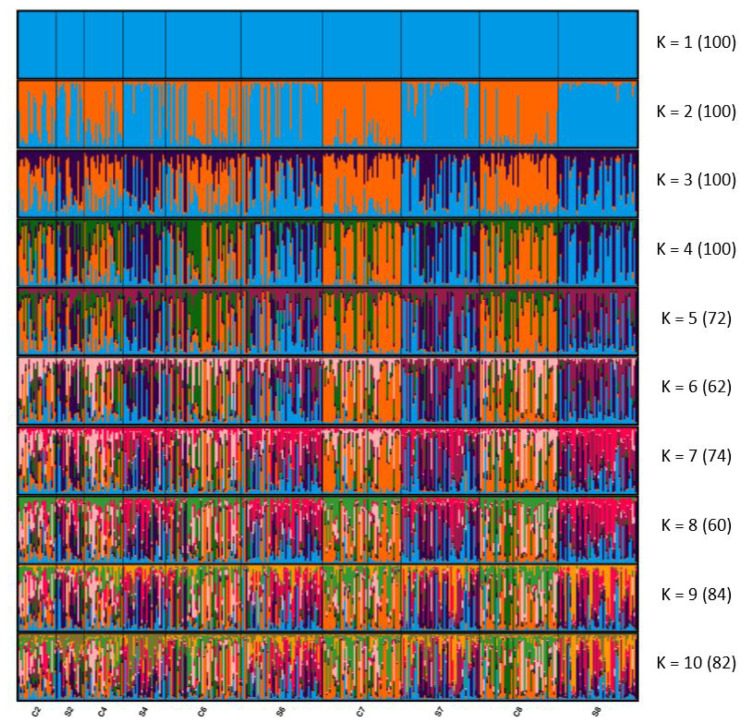
STRUCTURE clustering of the residual feed consumption (RFC) selected and control lines. Numbers in parentheses indicate the identical solutions of the 100 runs at the 95% threshold, and colors correspond to genetic clusters. Each vertical bar represents an individual. S, RFC selected line; C, the control line.

Figure 1 presents the results of the STRUCTURE analysis visualized using CLUMPAK 1.1. The optimal K was 2, estimated through the Evanno method [32]. When K was 2, although the genetic structure of the second and fourth generations slightly differed, mixed clusters were observed. After the seventh generation, the two lines formed distinct clusters. When K was 3, although the populations formed mixed clusters, the genetic structure differed slightly between C2 and C7 and between S2 and S7. In the eighth generation, the difference from the second generation is more obvious than the seventh generation. The STRUCTURE results indicate that the differentiation between the selected and control lines increased with each generation, similar to the *F*_ST_ and AMOVA results.

## DISCUSSION

### Microsatellite marker polymorphism

So far, FAO has not offered recommended microsatellite markers for ducks, so this study used the 11 microsatellite markers from Brown Tsaiya ducks [16] with superior polymorphisms. The average N_e_ of Korean and Bangladeshi ducks is 2.8 to 3.7 [34], and that of local duck breeds in India is 2 to 2.9 [5]. In this study, the average N_e_ of each generation of the two lines was 2.4 to 3.0, which is not significantly different from those observed in other studies.

To confirm the polymorphism of the markers used in this study, we also tested the 11 microsatellite markers in Pekin duck, Kaiya, and several lines of Brown and White Tsaiya ducks to investigate polymorphisms in other breeds. The mean N_a_ per locus was 7.3, and the mean N_e_ per locus was 3.6. The mean H_O_ was 0.474, the mean H_E_ was 0.690, and the mean PIC was 0.646. All markers were highly polymorphic, except for APT001 and APT032, which were moderately polymorphic [35]. Compared to our study, the study with the newly screened markers from Brown Tsaiya duck, there was an average N_a_ was 11.3 per locus, the mean N_e_ was 5.4, the mean H_O_ was 0.591, the mean H_E_ was 0.747, and the mean PIC was 0.708 [14]; the genetic variation was slightly higher than in this study.

Through the Ewens-Watterson test, we found that APT008 and APT026 were non-neutral markers. However, Pampoulie et al [36] pointed out that both neutral and non-neutral genetic markers gave consistent results, the overall level of genetic differentiation was similar whether or not the non-neutral marker was removed, even combined with non-neutral markers can provide better understand the potential effects of selection [36]. In this study, the purpose is to investigate the genetic variation and genetic structure of RFC selected and control lines, so the non-neutral markers were not excluded.

All markers used in this study were four-nucleotide repeats to prevent genotyping errors than the dinucleotide and trinucleotide repeats. The duck genome has been gradually revealed since 2020. The microsatellite libraries of the 11 markers were queried in the RefSeq duck (*A. platyrhynchos*) genome database (GCF_015476345.1_ZJU1.0) through BLAST to find possible chromosomal locations. The 11 markers were distributed on chromosomes 1, 2, 3, 7, and Z (Table 2). However, all 11 markers were on different duck chromosome scaffolds when we queried them in the database’s previous version (GCF_000355885.1), suggesting a considerable distance between them. Therefore, we considered that the 11 Tsaiya microsatellite markers had a substantial-resolution in the genetic analysis.

However, in five microsatellite markers, the number of alleles identified is equal or less than four. The low N_a_ may affect the estimation of differentiation level, diversities and heterozygosity. For example, markers with high N_a_ are more likely to show differences in allele frequencies among different population. Taking biallelic single nucleotide polymorphisms (SNPs) as an example, it is necessary to increase the number of markers by 2 to 11 times to achieve the same resolution of microsatellite markers [37]. Therefore, we will add highly polymorphic markers from other studies and consider the repeat motifs and positions on chromosomes in the future.

### Inter- and intra-line comparison of genetic variations

The results showed there were slightly decrease in the genetic variations, especially about the heterozygosity in the control line. Among them, the genetic variations of the second and fourth generations are slightly lower than those of the sixth generation. Hale et al [38] pointed out that when the sample size in a single population was less than 25, some alleles may not be detected, resulting in low N_a_, and the allele frequencies and H_E_ estimated by samples may be quite different from the real population. However, the sample size of 25 to 30 is representative, and it is little benefit of further increasing the sample size per population. Since the sample size in the second and fourth generations of this study is less than 30, the genetic variation may be underestimated [38]. For subsequent genetic monitoring, at least 30 individuals per population should be sampled.

And there was also increase in *F*_IS_ in the both line, although no consistent increase in *F*_IS_ was observed in each line. It indicated there were heterozygosity deficits in both the selected and control lines. Studies have indicated that possible causes of heterozygosity deficits include selection, inbreeding, geographic isolation, sampling error, the Wahlund effect, and null alleles [39].

The *F*_IS_ of each population was similar to the one of the total population (Tables 3 and 4). The ratio of the markers deviated from the Hardy–Weinberg equilibrium after the sixth generation in the two lines were 9% to 18% (1/11 to 2/11), which were less than the one in Lai et al [14] (7/24, 29%). The study also indicated H_O_ is less than H_E_ in Taiwan’s duck breeds and lines, even in the germplasm-conserved Brown Tsaiya duck and White Tsaiya duck with rotational mating schemes and without selection. It’s just the degree of heterozygosity deficit in these two conserved populations was lower than that in other selected populations. Also one study genetically monitored five Italian populations with *in situ* conservation and a circular mating scheme for four years. They discovered that *F*_IS_ decreased in all but one slightly selected population, in which *F*_IS_ increased significantly [40]. In our study, the selected line has been selected for RFC for more than ten years. Although the control line has not been selected for any additional trait, its ancestor, Brown Tsaiya LRI 1, has been selected for egg-laying performance for a long time. So selection should be one of the reasons for heterozygosity deficit in this study.

From the zero to eighth generations, the RFC selected line in each generation comprised 65 to 86 drakes, with a selection rate of 14% to 28.6%, and 104 to 152 female ducks, with a selection rate of 20.8% to 82.6%. The control line comprised 64 to 84 drakes and 89 to 144 female duck, with a selection rate of 14.1% to 18.8% and 27.8% to 51.7%, respectively (Supplementary Table S1). Only the zero generation was not divided into two lines, and the third generation of the selected line may have been affected by the quality of the feed, resulting in a smaller population than other generations [13]. Because the original populations sizes were small and genetic drift, low-frequency alleles were unlikely to be observed (for example, the 201 bp allele of APT012 only appeared in C2) and this increased the differences in H_O_, H_E_, and *F*_IS_ and decreased heter-ozygosity.

Although there were increase in *F*_IS_ in the both line, however, the *F*_IS_ was not significantly different from 0 and did not increase sharply. This may have been due to attempting to prevent full- and half-sib mating in both lines. The FreeNA results (Supplementary Table S4) showed that the frequencies of null allele in APT001 and APT033 were higher or close to 0.2 in more than half of the populations. The results of BLAST to RefSeq indicated the two markers located on sex chromosomes, and may result in PCR failure in one of an allele. We think it may cause part of the null allele. After the markers with null allele frequencies higher than 0.2 were excluded, except for S6 (no markers have been removed), the average *F*_IS_ of each population of the selected line and its control line was less than 0.1.

We also pay attention to the changes of allele, the number of private alleles mainly appeared in the selected line, which corresponds to Petit et al [41] consider private allele to contribute to response to selection or have evolutionary significance. And it may also make us to see higher N_a_ on the selected line. In the other hand, the AR in our study showed a downward trend over time, especially in the S8. AR is considered sensitive to founder events, compared to heterozygosity, AR decrease while the loss of alleles during founder events [42]. Therefore, the reason for the decline in AR should be mainly related to RFC selection.

Finally, to understand the status of genetic diversity and conservation management, we estimated the effective population size of the selected and control line. Few temporal sampling studies have been conducted, and most studies on Ne have used the linkage disequilibrium (LD) method, which is not strongly affected by genetic drift, resulting in higher Ne values. The LD method is limited by random mating and lifetime monogamy [24], which are inapplicable to the population in this experiment. Therefore, we only used a temporal method for estimation. The estimated Ne values of both lines in this experiment were 27.2 in the selected line and 23.9 in the control line. The Ne in the control (23.9) line was lower than the selected line (27.2) may be result from lower actual population size since the sixth generation (Supplementary Table S1). However, the Ne in both line were higher than Hungarian chicken breed study using a temporal method (4.4 to 12.7) [11]. It is similar to Speckled Hungarian in the same study and a study on indigenous Chinese chickens estimated by genome-wide SNPs using the LD method [43].

In summary, the heterozygosity deficit and change in *F*_IS_ each generation may have been caused by selection for RFC, genetic drift, and null alleles. Also, RFC selection may result in the decline in AR. However, the consistent *F*_IS_ over time, N_e_, and the performance monitoring results indicate no severe inbreeding depression. The follow-up genetic monitoring should be performed.

### Population differentiation and genetic structure analysis

According to Wright [44], an *F*_ST_ lower than 0.05 indicates nearly no differentiation, 0.05 to 0.15 indicates moderate differentiation, 0.15 to 0.25 indicates high differentiation, and values higher than 0.25 indicate extremely highly differentiation. Except for the sixth generation, the second through eighth generations was increasingly differentiated between the two lines (Table 5). As for inter-line pairwise *F*_ST_, the value between S6 and C6 indicated a decrease, followed by a gradual increase in the seventh and eighth generations. When we reproduced the fifth generation from the fourth generation, one of the drakes in the control line was mistakenly replaced with one from the selected line. The individuals with problematic pedigrees were gradually culled from the seventh and eighth generations. The temporary introduction of the selected line individual to the control line may have decreased the pairwise *F*_ST_ between S6 and C6 considerably, and have also caused the fluctuation in the *F*_IS_. When we culled the problematic individuals from the seventh and eighth generations, *F*_ST_ increased as the gap between generations widened, which can be attributed to the RFC selection for the selected line; the control line was not selected for RFC.

Although C6 was affected by the misuse of the selected line drakes, the increase in pairwise *F*_ST_ between generations in the control line may have been caused by the smaller population size and genetic drift. Similar to the genetic structure study of local chicken breeds in Hungary in 2002 and 2017 [11], the pairwise *F*_ST_ of the same breed at different times ranged from 0.03 to 0.08; this indicates slight differentiation. Because the populations in a Hungarian study were small, approximately 200 individuals, the differentiation may have also been caused by genetic drift.

The pairwise *F*_ST_ values of the seventh and eighth generations between the selected and control lines (i.e., Brown Tsaiya LRI 1) in this study were 0.1123 and 0.1271, respectively. Similar to the genetic analysis of Taiwan’s duck breeds and lines [14] indicated that the range of *F*_ST_ between Brown Tsaiya duck lines (preserved Brown Tsaiya duck, Brown Tsaiya LRI 1, LRI 2, and LRI 3) separated by more than ten generations was 0.092 to 0.167. The result indicated that the differentiation between the selected and control lines has reached the level of different lines. And the pairwise *F*_ST_ between lines from 0.0307 (C2 and S2) increased to 0.1271 (C8 and S8), the result is similar to Hungarian study. The pairwise *F*_ST_ between breeds ranged from 0.15 to 0.28 in 2002 and slightly increased to 0.21 to 0.30 after 15 years, the differentiation increased, and genetic similarity between breeds decreased over time [11].

*F*_IT_ (0.154) was mainly affected by *F*_IS_ (0.102) rather than by *F*_ST_ (Table 3). The AMOVA results for the second and eighth generations (Table 6) indicate that the primary genetic variation was between individuals. The variance ratio in the eighth generation is similar to that of other study on Brown Tsaiya ducks [14]. However, because the study included several lines of Brown Tsaiya ducks, the variance among the populations was slightly higher than that in this study. In this study, the variance among populations increased from 3% to 12% between the second and eighth generations, and the variance among individuals increased from 5% to 12%. The results are similar to those of the Hungarian study; the variance among populations increased from 23% to 25%, and the variance among individuals increased from 1% to 6% between 2002 and 2017 [11]. Therefore, the change in AMOVA results may also have been caused by genetic drift or selection.

As for PCA (Supplementary Figure S1), the first and second axes do not explain a considerable portion of the variation, and figure 1 indicates that each population did not form independent clusters. Our results are similar to those of Palinkas-Bodzsar et al [11], the distributions of the same breed sampled at an interval of 15 years were still very close, even though the first and second coordinates explained 9.13% and 17.47% of variations, higher than our study.

The STRUCTURE results are consistent with the pairwise *F*_ST_ results. When K was 2, the two lines formed independent clusters by the seventh generation. However, the proportions of inferred clusters on the left one-third of the samples in C6 were highly similar to the S6, and those in the other two-thirds of the samples were similar to C4. This may have been caused by the temporary introduced the selected line to the control line. Overall, the differentiation between the selected and control lines increased with each generation.

With the strengthening effects of climate change on the livestock industry, promoting “Better Feed Efficiency Brown Tsaiya” can help farmers reduce feed costs by 10% and mitigate the negative effects of extreme weather. However, maintaining large breeding populations is difficult with a limited workforce and management costs. Smaller poultry populations also often result in changes in allele frequency. Therefore, changes in heterozygosity over time must be monitored. Few studies have genetically monitored chickens over time, and no such study has been conducted on ducks. To ensure the sustainable management of key duck breeds, we used microsatellite markers to monitor ducks over time genetically. The results indicate that the markers reflect changes in heterozygosity and differentiation between the selected line and its control line. Although selection, genetic drift, and null alleles may have slightly decreased the heterozygosity and may result in the loss of allele in the two lines, heterozygosity remained, and *F*_IS_ did not increase considerably, indicating that the breeding and population management strategies were effective. To preserve genetic heterozygosity, rotational mating schemes, or increasing the number of drake families under the same population can be used if necessary. The results of this study indicate that genetic variation and structure did not change considerably after six generations with the current breeding and mating strategy and can be used as a reference for other poultry conservation research. Genetic monitoring should be performed regularly to conserve the germplasm, but the intervals between analyses can be longer to reduce costs.

## Figures and Tables

**Table 1:** The number of samples in each generation of RFC selected line and the control line used in this study

Generation	S		C	
	
	No. of drake	No. of duck	No. of drake	No. of duck
2	9	8	10	13
4	13	13	12	12
6	24	26	23	23
7	24	24	24	24
8	24	24	24	24

RFC, residual feed consumption; S, RFC selected line; C, the control line.

**Table 2:** Primer sequences, annealing temperature, and orthologous microsatellites in the duck genome scaffold of the 11 microsatellite markers derived from Tsaiya ducks

Locus^1)^	Primer sequences (5′→3′)	Ta (°C)^2)^	Chr.^3)^	Duck genome scaffold no.^4)^
APT001	F: GTCCCACTGGTTTGCTGTCC R: ACTACGCATGGCAGTGAGGTT	60	Z	1,509
APT004	F: GGGCAGGAAAATCTCCTGAAT R: TCTCAGTGGCTGAGCGGTC	60	3	192
APT008	F: CAAAGAAATCCTAGAACATCATTCAAAT R: TCTTCTGGCTTTTCACCTTAGTTTAGTA	60	1	358
APT010	F: CACTCAGGCTTTTAGGTCCATTAATA R: CATCTGAGAATGCACTTACTGTCAAA	60	2	1,199
APT012	F: TTGAGCCTCAGGTTCTAAACTCCTA R: TCATAACATTTCAGACCAGTTTTCAGA	60	2	5
APT017	F: TGGATGGACAGACGGGTGA R: TGGAAGTTTTGATTTCTAGTGCTTACA	60	1	481
APT020	F: TTCCAAGTTTGTCATGCCAATAGA R: CTGACCATGTTAGGGCGTTTTAG	60	1	197
APT025	F: TCCTAAGAAACGTTGCTTCATAGACC R: GAGTTAAGCTTCATCACTCTGTGACTG	60	2	121
APT026	F: CCCTGAAAGGCTGTTTTATATATCCA R: ATGTAAATAAAGTAGCCTTGCACGGT	60	7	477
APT032	F: TCACTTTCTTGACTCTCCTTGGTTT R: TGACTTGAATTCTGTTCAGGATAAATG	60	2	45
APT033	F: CTTCACCCTACCTCATAAGGAACTG R: ATTCCAAATCTGCAAGGTGAGTATTA	60	Z	14

1) Hsiao et al [16], developed from Tsaiya duck.

2) Annealing temperature.

3) Location on the duck chromosome.

4) Orthologous microsatellites in the duck genome scaffold.

**Table 3:** Characteristics of 11 Brown Tsaiya microsatellite markers used in five generations of RFC selected line and the control line

Items	Fragment (bp)	N_a_	N_e_	H_O_	H_E_	PIC	*F*_IS_	*F*_IT_	*F*_ST_
APT001^*^	174–206	4	2.389	0.315	0.582	0.497	0.454	0.471	0.032
APT004^*^	286–314	8	4.106	0.651	0.757	0.720	0.043	0.128	0.088
APT008^*^	184–196	4	3.579	0.446	0.722	0.672	0.331	0.391	0.089
APT010^*^	184–212	5	2.775	0.566	0.640	0.569	−0.034	0.103	0.133
APT012	181–205	6	3.534	0.674	0.718	0.665	0.009	0.066	0.058
APT017^*^	161–193	7	2.302	0.515	0.566	0.517	0.074	0.112	0.041
APT020	177–201	6	3.732	0.710	0.733	0.687	−0.031	0.019	0.048
APT025	105–121	5	1.977	0.505	0.495	0.441	−0.075	−0.036	0.036
APT026	130–146	4	3.684	0.758	0.730	0.678	−0.097	−0.064	0.030
APT032	207–259	3	1.913	0.475	0.478	0.381	−0.056	−0.035	0.021
APT033^*^	262–266	2	1.911	0.227	0.477	0.363	0.502	0.538	0.073
Average		4.909	2.900	0.531	0.627	0.563	0.102	0.154	0.059
SD		1.758	0.840	0.164	0.111	0.130	0.219	0.213	0.034

RFC, residual feed consumption; N_a_, number of observed alleles; N_e_, effective alleles; H_O_, observed heterozygosity, H_E_, expected heterozygosity; PIC, polymorphism information content; *F*_IS_, measure of the deviation from the Hardy–Weinberg proportions within subpopulation; *F*_IT_, measure of the deviation from Hardy–Weinberg model for total population; *F*_ST_, degree of differentiation between subpopulations; SD, standard deviation.

* Significant (p<0.01) deviation from Hardy–Weinberg equilibrium.

**Table 4:** Genetic variation analysis of the second, fourth, sixth, seventh, and eighth generations of RFC selected line and the control line with 11 Brown Tsaiya microsatellites markers

Line and generation^1)^	N	N_a_	N_e_	AR	N_p_	H_O_	H_E_	PIC	dHWE	*F*_IS_	Ne (CI)
S2	17	3.7	2.5	3.727	0.000	0.529	0.574	0.495	0	0.082±0.326	27.2 (17.3–63.4)
S4	26	3.9	2.4	3.686	0.182	0.503	0.571	0.494	0	0.135±0.250	
S6	50	4.1	2.7	3.680	0.091	0.531	0.609	0.538	2	0.134±0.207	
S7	48	4.1	2.4	3.543	0.364	0.496	0.574	0.503	1	0.137± 0.208	
S8	48	3.5	2.5	3.189	0.091	0.517	0.580	0.497	2	0.123±0.272	
C2	23	3.8	2.9	3.773	0.091	0.617	0.634	0.556	0	0.048±0.365	23.9 (16.6–42.3)
C4	24	3.7	2.9	3.649	0.000	0.530	0.635	0.554	0	0.165±0.319	
C6	46	3.9	3.0	3.777	0.000	0.550	0.636	0.567	2	0.141±0.225	
C7	48	3.9	2.6	3.719	0.000	0.561	0.595	0.530	0	0.084±0.208	
C8	48	3.6	2.5	3.545	0.000	0.504	0.571	0.509	2	0.104±0.252	

RFC, residual feed consumption; N, sample size; N_a_, number of observed alleles; N_e_, effective alleles; AR, allele richness; N_p_, number of private alleles per locus; H_O_, observed heterozygosity, H_E_, expected heterozygosity; PIC, polymorphism information content; dHWE, number of markers deviating from Hardy–Weinberg equilibrium; *F*_IS_, Wright’s fixation index, within population inbreeding estimate, and standard deviations; Ne (CI), effective population sizes with 95% jackknife confidence interval for second and eighth generations estimated using Jorde–Ryman temporal method.

1) S, RFC selected line; C, the control line; S6–S8, C6–C8 were genotyped in this study; other populations were genotyped in our previous study [13].

**Table 5:** *F*_ST_ values based on 11 Brown Tsaiya microsatellite markers for the second, fourth, sixth, seventh, and eighth generations of the RFC selected line and its control line

	S2^1)^	C4	S4	C6	S6	C7	S7	C8	S8
C2^1)^	0.0307	0.0054	0.0611^*^	0.0104	0.0332^*^	0.0387^*^	0.0649^*^	0.0606^*^	0.0738^*^
S2		0.0605^*^	0.0177	0.0340^*^	0.0287	0.0804^*^	0.0387	0.0945^*^	0.0510^*^
C4			0.0540^*^	0.0028	0.0405^*^	0.0276	0.0773^*^	0.0353^*^	0.0657^*^
S4				0.0428^*^	0.0191	0.1089^*^	0.0227	0.1172^*^	0.0127^*^
C6					0.0217	0.0191	0.0444^*^	0.0240^*^	0.0524^*^
S6						0.0768^*^	−0.0008	0.0875^*^	0.0074^*^
C7							0.1123^*^	−0.0008	0.1194^*^
S7								0.1235^*^	0.0085
C8									0.1271^*^

RFC, residual feed consumption.

1) S, RFC selected line; C, the control line; numbers of the populations indicate the generation.

* *F*_ST_ with significant differentiation (p<0.05).

**Table 6:** Summary of AMOVA results for second and eighth generations of the RFC selected line and its control line

Source	df		SS		MS		Est. Var.	%	Est. Var.	%
				
G2^1)^	G8^1)^	G2	G8	G2	G8	G2		G8	
Among populations	1	1	7.661	46.714	7.661	46.714	0.106	3	0.448	12
Among individuals	38	94	133.451	345.042	3.512	3.671	0.162	5	0.447	12
Within individuals	40	96	127.500	266.500	3.188	2.776	3.188	92	2.776	76
Total	79	191	268.613	658.255	-	-	3.456	100	3.676	100

AMOVA, analysis of molecular variance; RFC, residual feed consumption; df, degrees of freedom; SS, sum of squares; MS, mean squares; Est. Var., estimated variance, given in percentages.

1) G2, second generation of the RFC selected line and the control line; G8, eighth generation of the RFC selected line and the control line.

## SUPPLEMENTARY MATERIAL

**Supplementary Figure S1**. Principle coordinate analysis (PCA) plot of the low residual feed consumption (RFC) selected line and the control line based on allele frequencies of 11 Brown Tsaiya microsatellite markers.

**Supplementary Table S1**. RFC selected line and the control line populations

**Supplementary Table S2**. The Ewens–Watterson test for the selective neutrality of 11 Brown Tsaiya microsatellite markers by using Popgene

**Supplementary Table S3**. Effective population size (Ne) was estimated in two generations of each line using the Jordan–Ryman temporal method and plan I in NeEstimator

**Supplementary Table S4**. The Null allele frequencies ten populations with 11 Brown Tsaiya microsatellite markers

## References

[1] Liu HC, Tu TC, Marie-Etancelin C, Lee YP, Huang JF, Chen CF (2012). Genetic parameters of residual feed intake in the Brown Tsaiya duck. Taiwan Lives Res.

[2] FAO (2012). Phenotypic characterization of animal genetic resources. FAO Animal Production and Health Guidelines no 11.

[3] Magpantay VA, Lambio AL, Laude RP (2019). Genetic diversity of Philippine mallard duck (Anas platyrhynchos domesticus L.) based on SSR markers. Philipp J Sci.

[4] Pham LD, Do DN, Nam LQ (2022). Evaluation of genetic diversity and population structure in four indigenous duck breeds in Vietnam. Anim Biotechnol.

[5] Veeramani P, Prabakaran R, Sivaselvam SN (2021). Genetic diversity of six duck populations in India. Indian J Anim Res.

[6] Zhang X, He Y, Zhang W (2021). Development of microsatellite marker system to determine the genetic diversity of experimental chicken, duck, goose, and pigeon populations. Biomed Res Int.

[7] Zhang Y, Wang L, Bian Y (2019). Marginal diversity analysis of conservation of Chinese domestic duck breeds. Sci Rep.

[8] Gemayel R, Vinces MD, Legendre M (2010). Variable tandem repeats accelerate evolution of coding and regulatory sequences. Annu Rev Genet.

[9] Liu ZJ, Cordes JF (2004). DNA marker technologies and their applications in aquaculture genetics. Aquaculture.

[10] Tamaki K, Rapley R, Whitehouse D (2007). Minisatellite and microsatellite DNA typing analysis. Molecular forensics.

[11] Palinkas-Bodzsar N, Sztan N, Molnar T, Hidas A (2020). Gene conservation of six Hungarian local chicken breeds maintained in small populations over time. PloS one.

[12] Tu Y, Shu J, Ji G, Zhang M, Zou J (2018). Monitoring conservation effects on a Chinese indigenous chicken breed using major histocompatibility complex B-G gene and DNA Barcodes. Asian-Australas J Anim Sci.

[13] Chang YY, Chang WP, Wei LY (2018). Study on genetic structure in different Brown Tsaiya duck lines with microsatellite markers. J Chin Soc Anim Sci.

[14] Lai FY, Chang YY, Chen YC (2020). Monitoring of genetically close Tsaiya duck populations using novel microsatellite markers with high polymorphism. Asian-Australas J Anim Sci.

[15] Bush KL, Vinsky MD, Aldridge CL, Paszkowski CA (2005). A comparison of sample types varying in invasiveness for use in DNA sex determination in an endangered population of greater Sage-Grouse (Centrocercus uropihasianus). Conserv Genet.

[16] Hsiao MC, Liu HC, Hsu YC (2008). Isolation and characterization of microsatellite markers in Tsaiya duck. Asian-Australas J Anim Sci.

[17] Nei M (1978). Estimation of average heterozygosity and genetic distance from a small number of individuals. Genetics.

[18] Botstein D, White RL, Skolnick M, Davis RW (1980). Construction of a genetic linkage map in man using restriction fragment length polymorphisms. Am J Hum Genet.

[19] Kalinowski ST, Taper ML, Marshall TC (2007). Revising how the computer program cervus accommodates genotyping error increases success in paternity assignment. Mol Ecol.

[20] Yeh FC, Boyle TBJ (1997). Population genetic analysis of co-dominant and dominant markers and quantitative traits. Belg J Bot.

[21] Guo SW, Thompson EA (1992). Performing the exact test of Hardy-Weinberg proportion for multiple alleles. Biometrics.

[22] Manly BFJ (1985). The statistics of natural selection on animal populations.

[23] Chapuis MP, Estoup A (2007). Microsatellite null alleles and estimation of population differentiation. Mol Biol Evol.

[24] Do C, Waples RS, Peel D (2014). NeEstimator v2: re-implementation of software for the estimation of contemporary effective population size (Ne) from genetic data. Mol Ecol Resour.

[25] Jorde PE, Ryman N (2007). Unbiased estimator for genetic drift and effective population size. Genetics.

[26] Goudet J (c2001). FSTAT a program to estimate and test gene diversities and fixation indices (version 293) [Internet].

[27] Weir BS, Cockerham CC (1984). Estimating F-statistics for the analysis of population structure. Evolution.

[28] Peakall R, Smouse PE (2012). GenAlEx 6.5: genetic analysis in Excel. Population genetic software for teaching and research--an update. Bioinformatics.

[29] Jombart T, Ahmed I (2011). Adegenet 1.3-1: new tools for the analysis of genome-wide SNP data. Bioinformatics.

[30] Pritchard JK, Stephens M, Donnelly P (2000). Inference of population structure using multilocus genotype data. Genetics.

[31] Earl DA, vonHoldt BM (2012). STRUCTURE HARVESTER: a website and program for visualizing STRUCTURE output and implementing the Evanno method. Conserv Genet Resour.

[32] Evanno G, Regnaut S, Goudet J (2005). Detecting the number of clusters of individuals using the software STRUCTURE: a simulation study. Mol Ecol.

[33] Kopelman NM, Mayzel J, Jakobsson M, Rosenberg NA, Mayrose I (2015). Clumpak: a program for identifying clustering modes and packaging population structure inferences across K. Mol Ecol Resour.

[34] Sultana H, Seo D, Choi N (2017). Genetic diversity analyses of Asian duck populations using 24 microsatellite markers. Korean J Poult.

[35] Chang YY, Wei LY, Chen JY, Liu HC (2022). Study on genetic structure and phylogeny in Chihsin duck selected for the duration of fertility. Taiwan Lives Res.

[36] Pampoulie C, Daníelsdóttir AK, Storr-Paulsen M, Hovgård H, Hjörleifsson E, Steinarsson B (2011). Neutral and nonneutral genetic markers revealed the presence of inshore and offshore stock components of Atlantic cod in greenland waters. Trans Am Fish Soc.

[37] Zimmerman SJ, Aldridge CL, Oyler-McCance SJ (2020). An empirical comparison of population genetic analyses using microsatellite and SNP data for a species of conservation concern. BMC Genomics.

[38] Hale ML, Burg TM, Steeves TE (2012). Sampling for microsatellite-based population genetic studies: 25 to 30 individuals per population is enough to accurately estimate allele frequencies. PloS one.

[39] Dharmarajan G, Beatty WS, Rhodes OE (2013). Heterozygote deficiencies caused by a Wahlund effect: Dispelling unfounded expectations. J Wildl Manage.

[40] Zanetti E, De Marchi M, Abbadi M, Cassandro M (2011). Variation of genetic diversity over time in local Italian chicken breeds undergoing in situ conservation. Poult Sci.

[41] Petit RJ, El Mousadik A, Pons O (1998). Identifying populations for conservation on the basis of genetic markers. Conserv Biol.

[42] Manjula P, Kim M, Cho S, Seo D, Lee JH (2021). Highlevels of genetic variation in MHC-linked microsatellite markers from native chicken breeds. Genes.

[43] Zhang M, Han W, Tang H (2018). Genomic diversity dynamics in conserved chicken populations are revealed by genome-wide SNPs. BMC Genom.

[44] Wright S (1978). Evolution and the genetics of populations.

