# Supplementary material for: Monitoring changes in the genetic structure of Brown Tsaiya duck selected for feeding efficiency by microsatellite markers
Source: Anim Biosci. 2022 Nov 13;36(3):417–28. doi: 10.5713/ab.22.0213 (PMC9996257; doi:10.5713/ab.22.0213)
Supplement: Supplementary file 1 [file ab-22-0213-Supplementary-Fig-1.pdf]

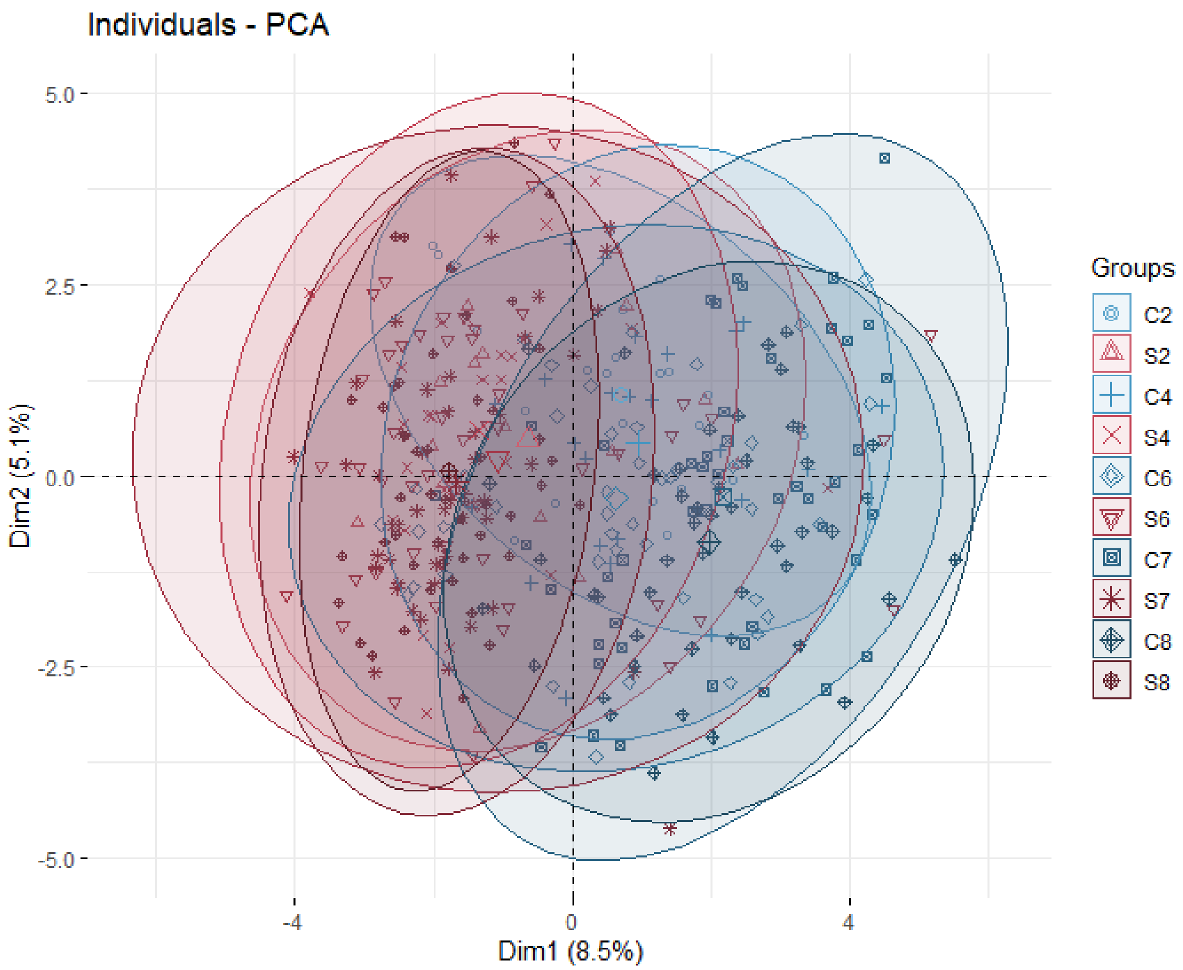

Supplementary Figure S1. Principle coordinate analysis (PCA) plot of the RFC selected line and the control line based on allele frequencies of 11 Brown Tsaiya microsatellite markers. The first (PC1), second (PC2) principal components account for 8.5% and 5.2% of the total variation, respectively. S: RFC selected line; C: the control line.
