# Supplementary material for: Monitoring changes in the genetic structure of Brown Tsaiya duck selected for feeding efficiency by microsatellite markers
Source: Anim Biosci. 2022 Nov 13;36(3):417–28. doi: 10.5713/ab.22.0213 (PMC9996257; doi:10.5713/ab.22.0213)
Supplement: Supplementary file 2 [file ab-22-0213-Supplementary-Table-1.pdf]

Supplementary Table S1. RFC selected line and the control line populations

Generation.	Line	No. of individuals	No. of breeders	Selection rate (%)
0		M = 157	M = 20	12.7
		F = 195	F = 82	42.1
1	S	M = 66	M = 10	15.2
		F = 119	F = 42	35.3
	C	M = 78	M = 11	14.1
		F = 120	F = 46	38.3
2	S	M = 70	M = 12	17.1
		F = 125	F = 26	20.8
	C	M = 83	M = 12	14.5
		F = 123	F = 61	49.6
3	S	M = 42	M = 12	28.6
		F = 46	F = 38	82.6
	C	M = 84	M = 12	14.3
		F = 89	F = 46	51.7
4	S	M = 65	M = 11	16.9
		F = 104	F = 42	40.4
	C	M = 81	M = 13	16.0
		F = 118	F = 46	39.0
5	S	M = 71	M = 12	16.9
		F = 136	F = 40	29.4
	C	M = 82	M = 12	14.6
		F = 144	F = 40	27.8
6	S	M = 78	M = 12	15.4
		F = 148	F = 45	30.4
	C	M = 64	M = 12	18.8
		F = 94	F = 46	48.9
7	S	M = 86	M = 12	14.0
		F = 126	F = 45	35.7
	C	M = 74	M = 12	16.2
		F = 94	F = 43	45.7
8	S	M = 80	M = 12	15.0
		F = 152	F = 45	29.6
	C	M = 76	M = 12	15.8
		F = 113	F = 43	38.1

S: RFC selected line; C: the control line.
