# Supplementary material for: Monitoring changes in the genetic structure of Brown Tsaiya duck selected for feeding efficiency by microsatellite markers
Source: Anim Biosci. 2022 Nov 13;36(3):417–28. doi: 10.5713/ab.22.0213 (PMC9996257; doi:10.5713/ab.22.0213)
Supplement: Supplementary file 3 [file ab-22-0213-Supplementary-Table-2.pdf]

Supplementary Table S2. The Ewens–Watterson test for the selective neutrality of 11 Brown Tsaiya microsatellite markers by using Popgene.

Locus	Obs. F	SE	L95	U95
APT01	0.4186	0.0384	0.3367	0.9788
APT04	0.2435	0.0250	0.2258	0.8330
APT08	0.2794*	0.0393	0.3301	0.9812
APT10	0.3604	0.0379	0.2955	0.9608
APT12	0.2830	0.0326	0.2629	0.9131
APT17	0.4343	0.0298	0.2360	0.8909
APT20	0.2680	0.0355	0.2590	0.9379
APT25	0.5059	0.0366	0.3062	0.9504
APT26	0.2714*	0.0391	0.3455	0.9789
APT32	0.5228	0.0372	0.3878	0.9916
APT33	0.5234	0.0270	0.5064	0.9973

The statistics were calculated using 1000 simulated samples.

* Significant ($P < 0.01$) deviation from neutrality; Obs. F, observed sum of the square of allelic frequency; SE, Standard error of the mean; L95, Lower 95% confidence limit; U95, Upper 95% confidence limit.
