# Supplementary material for: Monitoring changes in the genetic structure of Brown Tsaiya duck selected for feeding efficiency by microsatellite markers
Source: Anim Biosci. 2022 Nov 13;36(3):417–28. doi: 10.5713/ab.22.0213 (PMC9996257; doi:10.5713/ab.22.0213)
Supplement: Supplementary file 4 [file ab-22-0213-Supplementary-Table-3.pdf]

Supplementary Table S3. Effective population size (Ne) was estimated in two generations of each line using the Jordan–Ryman temporal method and plan I in NeEstimator

Line N	Pop pair	Mean size	Ne	CI	
				Lower	Upper
195	S2<->S4	20.6	21.9	8.5	Infinite
195	S2<->S6	25.2	29.6	15.2	581.5
195	S2<->S7	25	29.0	16.1	142.8
195	S2<->S8	25	27.2	17.3	63.4
195	S4<->S6	33.9	21.3	11.2	204.1
195	S4<->S7	33.6	27.5	15.5	121
195	S4<->S8	33.6	60.2	34	262
195	S6<->S7	48	85.8	28.2	Infinite
195	S6<->S8	48.1	43.9	24.5	215.6
195	S7<->S8	47.6	20.0	9.6	Infinite
206	C2<->C4	23.5	50.0	19.2	Infinite
206	C2<->C6	30.5	71.7	38.6	504.1
206	C2<->C7	31	31.0	20.3	65.8
206	C2<->C8	30.9	23.9	16.6	42.3
206	C4<->C6	31.3	66.2	33	Infinite
206	C4<->C7	31.9	24.3	13.3	142.2
206	C4<->C8	31.8	25.8	14.6	112.3
206	C6<->C7	46.4	11.1	7	26.6
206	C6<->C8	46	18.0	10.8	53.1
206	C7<->C8	47.3	93.1	33.6	Infinite

Line N, sample size in each line; Pop pair, Populations to be compared; Mean size, the average sample size of the compared two populaitons; CI, 95% jackknife confidence interval.
