# Supplementary material for: Monitoring changes in the genetic structure of Brown Tsaiya duck selected for feeding efficiency by microsatellite markers
Source: Anim Biosci. 2022 Nov 13;36(3):417–28. doi: 10.5713/ab.22.0213 (PMC9996257; doi:10.5713/ab.22.0213)
Supplement: Supplementary file 5 [file ab-22-0213-Supplementary-Table-4.pdf]

Supplementary Table S4. The Null allele frequencies ten populations with 11 Brown Tsaiya microsatellite markers

	C2	S2	C4	S4	C6	S6	C7	S7	C8	S8
APT001	0.20	0.24	0.10	0.15	0.22	0.17	0.07	0.22	0.19	0.11
APT004	0.00	0.03	0.11	0.00	0.04	0.07	0.04	0.02	0.08	0.00
APT008	0.00	0.10	0.35	0.24	0.12	0.11	0.05	0.08	0.25	0.20
APT010	0.00	0.00	0.00	0.05	0.01	0.05	0.00	0.02	0.03	0.00
APT012	0.01	0.00	0.04	0.00	0.00	0.00	0.00	0.11	0.00	0.00
APT017	0.17	0.00	0.11	0.00	0.00	0.01	0.05	0.04	0.03	0.00
APT020	0.01	0.00	0.00	0.00	0.01	0.04	0.00	0.00	0.00	0.00
APT025	0.00	0.00	0.00	0.00	0.00	0.00	0.00	0.00	0.00	0.09
APT026	0.00	0.00	0.00	0.00	0.00	0.00	0.00	0.06	0.00	0.00
APT032	0.00	0.00	0.00	0.04	0.00	0.00	0.07	0.06	0.00	0.01
APT033	0.24	0.20	0.17	0.12	0.17	0.18	0.15	0.10	0.04	0.19

S: RFC selected line; C: the control line; Numbers of the populations indicate the generation.
